# Supplementary material for: Two NLR immune receptors acquired high-affinity binding to a fungal effector through convergent evolution of their integrated domain
Source: eLife. 2021 Jul 21;10:e66961. doi: 10.7554/eLife.66961 (PMC8294853; doi:10.7554/eLife.66961)
Supplement: Supplementary file 1. [file elife-66961-supp1.docx]

| Species | Cultivar | Version | Accession no | Source | Reference |
| --- | --- | --- | --- | --- | --- |
| *Brachypodium distachyon* | Bd21 | v3.0 | GCA_000005505.4 | Ensembl Plants | International Brachypodium Initiative, 2010 |
| *Hordeum vulgare* | Morex | v2 | GCA_901482405.1 | Phytozome v12.1 | Mascher et al., 2017 |
| *Oryza brachyantha* | IRGC101232 | v1.4b | GCA_000231095.2 | Ensembl Plants | Chen et al., 2013 |
| *Oryza sativa* | Nipponbare | IRGSP-1.0 | GCA_001433935.1 | Ensembl Plants | Kawahara et al. 2013 |
| *Setaria italica* | Yugu1 | v2.2 | AGNK01000000.1 | Phytozome v12.1 | Bennetzen et al., 2012 |
| *Sorghum bicolor* | BTx623 | v3 | GCA_000003195.3 | Ensembl Plants | Paterson et al., 2009 |
| *Triticum aestivum* | Chinese Spring | v1.0 | GCA_900519105.1 | Ensembl Plants | International Wheat Genome Sequencing Consortium et al., 2018 |
| *Zea mays* | B73 | v4 | GCA_000005005.6 | Ensembl Plants | Zhang et al., 2009 |

**Supplementary file 1A. List of databases used for NLR identification.**

**Supplementary file 1B. List of known and functionally characterized NLR-type resistance proteins from grasses used as reference sequences.**

| Name | Accession number | Species | Reference |
| --- | --- | --- | --- |
| MLA10 | AY266445.1 | *Hordeum vulgare* | Halterman and Wise, 2004 |
| RGA1-A | KT725812.1 | *Secale cereale* | Mago et al., 2015 |
| Os11gRGA5 | AB604627.1 | *Oryza sativa* | Okuyama et al., 2011 |
| Os11gRGA4 | AB604622.1 | *Oryza sativa* | Okuyama et al., 2011 |
| Piz-t | DQ352040.1 | *Oryza sativa* | Zhou et al., 2006 |
| Pi-ta | AF207842.1 | *Oryza sativa* | Bryan et al., 2000 |
| Rpg5 | EU883792.1 | *Hordeum vulgare* | Brueggeman et al., 2008 |
| LR10 | AY270157.1 | *Triticum aestivum* | Feuillet et al., 2003 |
| Yr10 | AF149112.1 | *Triticum aestivum* | Liu et al., 2014 |
| Pib | AB013448.1 | *Oryza sativa* | Wang et al., 1999 |
| Pi9 | DQ285630.1 | *Oryza sativa* | Qu et al., 2006 |
| Rp1-D | XM_008664205.2 | *Zea mays* | Collins et al., 1999 |
| Xa1 | AB002266.1 | *Oryza sativa* | Yoshimura et al., 1998 |
| Pm8 | KF572030.1 | *Triticum aestivum* | Hurni et al., 2013 |
| Pm3 | GU230859.1 | *Triticum aestivum* | Bhullar et al., 2010 |
| Rdg2-a | HM124452.1 | *Hordeum vulgare* | Bulgarelli et al., 2010 |
| Lr21 | FJ876280.1 | *Triticum aestivum* | Huang et al., 2009 |
| Pit | AB379815.1 | *Oryza sativa* | Hayashi and Yoshida, 2009 |
| Pi5-1 | EU869185.1 | *Oryza sativa* | Lee et al., 2009 |
| Pi5-2 | EU869186.1 | *Oryza sativa* | Lee et al., 2009 |
| Pid3 | KX791058.1 | *Oryza sativa* | Shang et al., 2009 |
| Sr45 | LN883757.1 | *Triticum aestivum* | Steuernagel et al., 2016 |
| Sr22 | LN883743.1 | *Triticum aestivum* | Steuernagel et al., 2016 |
| Lr22a | KY064064.1 | *Triticum aestivum* | Thind et al., 2017 |
| Pik*-1 | HM048900_1 | *Oryza sativa* | Zhai et al., 2011 |
| Pik*-2 | ADZ48538.1 | *Oryza sativa* | Zhai et al., 2011 |
| Pikh-1 | HQ662330_1 | *Oryza sativa* | Costanzo and Jia, 2010b |
| Pikh-2 | AET36550.1 | *Oryza sativa* | Costanzo and Jia, 2010b |
| Pikm-1 | AB462324_1 | *Oryza sativa* | Ashikawa et al., 2008 |
| Pikm-2 | BAG72135.1 | *Oryza sativa* | Ashikawa et al., 2008 |
| Piks-1 | HQ662329_1 | *Oryza sativa* | Jia et al., 2009 |
| Piks-2 | AET36548.1 | *Oryza sativa* | Jia et al., 2009 |
| Pikp-1 | HM035360.1 | *Oryza sativa* | Yuan et al., 2011 |
| Pikp-2 | ADV58351.1 | *Oryza sativa* | Yuan et al., 2011 |

| Accession | Country of origin | Used for *Pik-2* cloning | Used for *Pik-1* cloning |
| --- | --- | --- | --- |
| W0654 | Sierra Leone | Full-length | Full-length |
| W0655 | Sierra Leone | Not sequenced | Full-length |
| W0656 | Guinea | Fragment | Not amplified |
| W1057 | Guinea | Fragment | Not amplified |
| W1401 | Sierra Leone | Fragment | Not amplified |
| W1402 | Sierra Leone | Fragment | Not amplified |
| W1403 | Sierra Leone | Not sequenced | Not amplified |
| W1404 | Sierra Leone | Full-length | Full-length |
| W1405 | Sierra Leone | Full-length | Full-length |
| W1407(B) | Mali | Full-length | Full-length |
| W1703 | Mali | Full-length | Full-length |
| W1705 | Mali | Full-length | Full-length |
| W1706 | Chad | Fragment | Not amplified |
| W1708 | Cameroon | Fragment | Not amplified |
| W1711 | Cameroon | Fragment | Not amplified |
| W1712 | Cameroon | Fragment | Not amplified |

**Supplementary file 1C. List of *Oryza brachyantha* accessions.**

| Species | Version | Assembly accession number | Source | References |
| --- | --- | --- | --- | --- |
| *Dactylis glomerata* | v1 | GCA_007115705.1 | NCBI | Huang et al., 2020 |
| *Leersia perrieri* | v1.4 | GCA_000325765.3 | Ensembl Plants | Direct submission |
| *Oryza barthii* | v1 | GCA_003020155.1 | Ensembl Plants | Direct submission |
| *Oryza glaberrima* | v1 | GCA_000147395.2 | Ensembl Plants | Direct submission |
| *Oryza glumaepatula* | v1.5 | GCA_000576495.1 | Ensembl Plants | Direct submission |
| *Oryza longistaminata* | v1.0 | GCA_000789195.1 | Ensembl Plants | Direct submission |
| *Oryza nivara* | v1.0 | GCA_000576065.1 | Ensembl Plants | Direct submission |
| *Oryza punctata* | v1.2 | GCA_000573905.1 | Ensembl Plants | Direct submission |
| *Oryza rufipogon* | v1 | GCA_000817225.1 | Ensembl Plants | Direct submission |
| *Zizania latifolia* | v1 | GCA_000418225.1 | NCBI | Direct submission |

**Supplementary file 1D. List of plant datasets used for BLASTN search.**

| *Os* | *Oryza sativa* cv. K60 | Chromosome 11 | 27973977 | 28008157 |
| --- | --- | --- | --- | --- |
| *Oniv* | *Oryza nivara* | Chromosome 11 | 23864010 | 23917629 |
| *Oglum* | *Oryza glumaepatula* | Chromosome 11 | 26116594 | 26116594 |
| *Ol* | *Oryza longistaminata* | Contig CM003669.1 | 2965866 | 8003128 |
| *Opunc* | *Oryza punctata* | Chromosome 11 | 4972847 | 4983002 |
| *Ob* | *Oryza brachyantha* | Chromosome 11 | 15529280 | 15547390 |
| *Lp* | *Leersia perrieri* | Chromosome 11 | 20337647 | 20286182 |
| *Ta* | *Triticum aestivum* | Chromosome 1D | 33124348 | 31125148 |
| *Dg* | *Dactylis glomerata* | Scaffold QXEO01001682.1 | 1295679 | 1340805 |
| *Si* | *Setaria italica* | Scaffold 8 | 39159743 | 39261506 |
| *Sb* | *Sorghum bicolor* | Chromosome 2 | 6043453 | 6215456 |

**Supplementary file 1E.** **Coordinates of genomic regions used in** **Figure 1B.**

| *Triticum aestivum* | Chromosome 1D | 33124348 | 31125148 |
| --- | --- | --- | --- |
| *Triticum aestivum* | Chromosome 4A | 739372651 | 742475497 |
| *Triticum aestivum* | Chromosome 7D | 3066514 | 4338541 |
| *Sorghum bicolor* | Chromosome 5 | 70290712 | 70702146 |
| *Sorghum bicolor* | Chromosome 2 | 5911252 | 6248983 |
| *Setaria italica* | Scaffold 8 | 39100454 | 39349775 |

**Supplementary file 1F.** **Coordinates of genomic regions used in Figure 1 – Figure Supplement 4.**

| Name | Pik-1 | Pik-2 | Species |
| --- | --- | --- | --- |
| *Os*Pikp | Pikp-1_HM035360.1 | Pikp-2_HM035360.1 | *Oryza sativa* |
| *Os*Pikh | Pikh-1_HQ662330.1 | Pikh-2_HQ662330.1 | *Oryza sativa* |
| *Os*Pik* | Pik*-1_HM048900_1 | Pik*-2_HM048900_1 | *Oryza sativa* |
| *Os*Piks | Piks-1_HQ662329_1 | Piks-2_HQ662329_1 | *Oryza sativa* |
| *Os*Pikm | Pikm-1_BAG72135.1 | Pikm-2_BAG72135.1 | *Oryza sativa* |
| *Obart* Pik | OBART11G23150 | OBART11G23160 | *Oryza barthii* |
| *Olongi* Pik | KN541092.1_2 | KN541092.1 | *Oryza longistaminata* |
| *Opunc* Pik | OPUNC11G19550.n | OPUNC11G19560 | *Oryza punctata* |
| *Ob*Pik W1703 | ObPik-1_W1703 | ObPik-2_W1703 | *Oryza brachyantha* |
| *Ob*Pik W1407 | ObPik-1_W1407 | ObPik-2_W1407 | *Oryza brachyantha* |
| *Ob*Pik W1705 | ObPik-1_W1705 | ObPik-2_W1705 | *Oryza brachyantha* |
| *Ob*Pik IRGC101232 | OB11G27420.n | OB11G27420 | *Oryza brachyantha* |
| *Ob*Pik W1405 | ObPik-1_W1405 | ObPik-2_W1405 | *Oryza brachyantha* |
| *Ob*Pik W1404 | ObPik-1_W0654 | ObPik-2_W0654 | *Oryza brachyantha* |
| *Ob*Pik W0654 | ObPik-1_W1404 | ObPik-2_W1404 | *Oryza brachyantha* |
| *Oglum* Pik | OGLAB11G20210.1n | ORGLA11G0185700 | *Oryza glaberrima* |
| *Oglab* Pik | OGLUM11G22320.n | OGLUM11G22330 | *Oryza glumaepatula* |
| *Oniv* Pik | ORUFI11G24730 | ORUFI11G24740 | *Oryza rufipogon* |
| *Oruf* Pik | ONIVA11G22690.n | ONIVA11G22700 | *Oryza nivara* |
| *Os*Pik Nipp | Pikm5_NP Nipp_DP000010.2 | PIK6_NP XM_015762499.2 | *Oryza sativa* cv. Nipponbare |
| *Ta*Pik 1D | TraesCS1D02G051500.1 | TraesCS1D02G051400.1 | *Triticum aestivum* |
| *Sb*Pik 5 | SORBI_3005G219700 | SORBI_3005G219900 | *Sorghum bicolor* |
| *Si*Pik 8.1 | Seita.8G239300.n | Seita.8G239400 | *Setaria italica* |
| *Si*Pik 8.2 | Seita.8G238800 | Seita.8G238900 | *Setaria italica* |
| *Dg*Pik | QXEO01001682.1 | QXEO01001682.1_2 | *Dactylis glomerata* |
| *Ta*Pik 4A.1 | TraesCS4A02G493400.1 | TraesCS7A02G006200.1 | *Triticum aestivum* |
| *Ta*Pik 4A.2 | TraesCS4A02G491000.1 | TraesCS4A02G490900.1 | *Triticum aestivum* |
| *Ta*Pik 7D | TraesCS7D02G007700.1 | TraesCS7D02G007600.1 | *Triticum aestivum* |

**Supplementary file 1G. Genes used for the comparisons of *d*_S_ and rates of Pik-1–Pik-2 presented in** **Figure 1C.**

**Supplementary file 1H.** **Summary of the amplification experiment of the Pik-1–integrated HMA domain from wild rice species.**

| Accession | Species | Origin | Amplified | Sequence confirmed |
| --- | --- | --- | --- | --- |
| W0654 | *O. brachyantha* | Sierra Leone | Yes | Yes |
| W0008 | *O. australiensis* | Australia (SE Canberra) | Yes | No |
| W1628 | *O. australiensis* | Australia (N) | No | NA |
| W1643 | *O. barthii* | Botswana | Yes | Yes |
| W1605 | *O. barthii* | Nigeria | Yes | No |
| W0042 | *O. barthii* | unspecified | Yes | Yes |
| W0698 | *O. barthii* | Guinea | Yes | Yes |
| W1526 | *O. eichingeri* | Uganda | No | NA |
| W1171 | *O. glumaepatula* | Cuba | No | NA |
| W2203 | *O. glumaepatula* | Brazil (S) | Yes | No |
| W1480(B) | *O. grandiglumis* | Brazil (N) | Yes | No |
| W0005 | *O. granulata* | Sri Lanka | No | NA |
| W0067(B) | *O. granulata* | Thailand | Yes | Yes |
| W0542 | *O. latifolia / O. alta* | Mexico | No | NA |
| W1539 | *O. latifolia / O. alta* | Argentina (N) | No | NA |
| W1228 | *O. longiglumis* | Singapore (S) | No | NA |
| W1504 | *O. longistaminata* | Tanzania | No | NA |
| W1540 | *O. longistaminata* | Republic of Congo | Yes | Yes |
| W0643 | *O. longistaminata* | The Gambia | Yes | Yes |
| W2081 | *O. meridionalis* | Australia (N) | No | NA |
| W2112 | *O. meridionalis* | Australia (NE) | No | NA |
| W1354 | *O. meyeriana* | Malaysia | Yes | No |
| W1328 | *O. minuta* | Philippines | Yes | Yes |
| W0614 | *O. officinalis* | Myanmar | Yes | Yes |
| W1200 | *O. officinalis* | Philippines | Yes | Yes |
| W1408 | *O. punctata* | Nigeria | Yes | Yes |
| W1514 | *O. punctata* | Kenya | Yes | Yes |
| W1808 | *O. rhizomatis* | Sri Lanka | Yes | No |
| W0001 | *O. ridleyi* | Thailand | Yes | No |
| W2035 | *O. ridleyi* | Philippines | No | NA |
| W2003 | *O. rufipogon* | India (SW) | Yes | Yes |
| W1715 | *O. rufipogon* | Chin (Beijing) | No | NA |
| W2117 | *O. rufipogon/ O. meridionalis* | Australia (NE) | Yes | No |

**Supplementary file 1I. Table of p-values for all pairwise comparisons of SPR binding to AVR-PikD between the HMA mutants.**

| Concentration  (nM) | Sample 1 | Sample 2 | Difference | Lower  confidence level | Upper  confidence level | p-value |
| --- | --- | --- | --- | --- | --- | --- |
| 50 | IAQVV | E230R | 0.7594754 | -6.380544 | 7.899495 | 0.9999725 |
| 50 | LAKIE | E230R | 15.1954147 | 8.055395 | 22.335434 | 0.0000005 |
| 50 | LAKIV | E230R | 0.1973327 | -6.942687 | 7.337352 | 1 |
| 50 | LAKVV | E230R | -1.2876815 | -8.427701 | 5.852338 | 0.9990674 |
| 50 | LAQVV | E230R | -0.2110851 | -7.351104 | 6.928934 | 1 |
| 50 | LVKIE | E230R | 29.9244059 | 22.784387 | 37.064425 | 0 |
| 50 | Pikp-HMA | E230R | 21.3309677 | 14.190948 | 28.470987 | 0 |
| 50 | LAKIE | IAQVV | 14.4359393 | 7.29592 | 21.575959 | 0.0000016 |
| 50 | LAKIV | IAQVV | -0.5621428 | -7.702162 | 6.577877 | 0.9999965 |
| 50 | LAKVV | IAQVV | -2.047157 | -9.187176 | 5.092862 | 0.9838216 |
| 50 | LAQVV | IAQVV | -0.9705606 | -8.11058 | 6.169459 | 0.9998555 |
| 50 | LVKIE | IAQVV | 29.1649305 | 22.024911 | 36.30495 | 0 |
| 50 | Pikp-HMA | IAQVV | 20.5714923 | 13.431473 | 27.711512 | 0 |
| 50 | LAKIV | LAKIE | -14.998082 | -22.138101 | -7.858063 | 0.0000007 |
| 50 | LAKVV | LAKIE | -16.483096 | -23.623116 | -9.343077 | 0.0000001 |
| 50 | LAQVV | LAKIE | -15.4065 | -22.546519 | -8.266481 | 0.0000004 |
| 50 | LVKIE | LAKIE | 14.7289911 | 7.588972 | 21.86901 | 0.000001 |
| 50 | Pikp-HMA | LAKIE | 6.1355529 | -1.004466 | 13.275572 | 0.1407186 |
| 50 | LAKVV | LAKIV | -1.4850142 | -8.625033 | 5.655005 | 0.9976792 |
| 50 | LAQVV | LAKIV | -0.4084178 | -7.548437 | 6.731601 | 0.9999996 |
| 50 | LVKIE | LAKIV | 29.7270732 | 22.587054 | 36.867093 | 0 |
| 50 | Pikp-HMA | LAKIV | 21.133635 | 13.993616 | 28.273654 | 0 |
| 50 | LAQVV | LAKVV | 1.0765964 | -6.063423 | 8.216616 | 0.9997116 |
| 50 | LVKIE | LAKVV | 31.2120874 | 24.072068 | 38.352107 | 0 |
| 50 | Pikp-HMA | LAKVV | 22.6186492 | 15.47863 | 29.758669 | 0 |
| 50 | LVKIE | LAQVV | 30.135491 | 22.995472 | 37.27551 | 0 |
| 50 | Pikp-HMA | LAQVV | 21.5420528 | 14.402034 | 28.682072 | 0 |
| 50 | Pikp-HMA | LVKIE | -8.5934382 | -15.733457 | -1.453419 | 0.008645 |
| 200 | IAQVV | E230R | 8.12408613 | 4.769044 | 11.4791283 | 0 |
| 200 | LAKIE | E230R | 43.4535131 | 40.098471 | 46.8085553 | 0 |
| 200 | LAKIV | E230R | 7.26935912 | 3.914317 | 10.6244013 | 0.0000006 |
| 200 | LAKVV | E230R | -0.0357561 | -3.390798 | 3.3192861 | 1 |
| 200 | LAQVV | E230R | 4.96818917 | 1.613147 | 8.3232314 | 0.0006703 |
| 200 | LVKIE | E230R | 57.0910393 | 53.735997 | 60.4460815 | 0 |
| 200 | Pikp-HMA | E230R | 53.8341313 | 50.479089 | 57.1891735 | 0 |
| 200 | LAKIE | IAQVV | 35.329427 | 31.974385 | 38.6844692 | 0 |
| 200 | LAKIV | IAQVV | -0.854727 | -4.209769 | 2.5003152 | 0.9912868 |
| 200 | LAKVV | IAQVV | -8.1598423 | -11.514884 | -4.8048001 | 0 |
| 200 | LAQVV | IAQVV | -3.155897 | -6.510939 | 0.1991452 | 0.0782897 |
| 200 | LVKIE | IAQVV | 48.9669532 | 45.611911 | 52.3219953 | 0 |
| 200 | Pikp-HMA | IAQVV | 45.7100452 | 42.355003 | 49.0650873 | 0 |
| 200 | LAKIV | LAKIE | -36.184154 | -39.539196 | -32.829112 | 0 |
| 200 | LAKVV | LAKIE | -43.489269 | -46.844311 | -40.134227 | 0 |
| 200 | LAQVV | LAKIE | -38.485324 | -41.840366 | -35.130282 | 0 |
| 200 | LVKIE | LAKIE | 13.6375262 | 10.282484 | 16.9925683 | 0 |
| 200 | Pikp-HMA | LAKIE | 10.3806182 | 7.025576 | 13.7356603 | 0 |
| 200 | LAKVV | LAKIV | -7.3051152 | -10.660157 | -3.9500731 | 0.0000006 |
| 200 | LAQVV | LAKIV | -2.30117 | -5.656212 | 1.0538722 | 0.3776686 |
| 200 | LVKIE | LAKIV | 49.8216802 | 46.466638 | 53.1767223 | 0 |
| 200 | Pikp-HMA | LAKIV | 46.5647722 | 43.20973 | 49.9198144 | 0 |
| 200 | LAQVV | LAKVV | 5.00394529 | 1.648903 | 8.3589875 | 0.0006038 |
| 200 | LVKIE | LAKVV | 57.1267954 | 53.771753 | 60.4818376 | 0 |
| 200 | Pikp-HMA | LAKVV | 53.8698874 | 50.514845 | 57.2249296 | 0 |
| 200 | LVKIE | LAQVV | 52.1228501 | 48.767808 | 55.4778923 | 0 |
| 200 | Pikp-HMA | LAQVV | 48.8659421 | 45.5109 | 52.2209843 | 0 |
| 200 | Pikp-HMA | LVKIE | -3.256908 | -6.61195 | 0.0981342 | 0.0625642 |
| 400 | IAQVV | E230R | 15.4160802 | 10.476265 | 20.3558957 | 0 |
| 400 | LAKIE | E230R | 59.1376719 | 54.197856 | 64.0774874 | 0 |
| 400 | LAKIV | E230R | 17.2208188 | 12.281003 | 22.1606344 | 0 |
| 400 | LAKVV | E230R | -0.6186041 | -5.55842 | 4.3212114 | 0.9999089 |
| 400 | LAQVV | E230R | 10.5836822 | 5.643867 | 15.5234977 | 0.0000008 |
| 400 | LVKIE | E230R | 67.4451738 | 62.505358 | 72.3849893 | 0 |
| 400 | Pikp-HMA | E230R | 66.0719577 | 61.132142 | 71.0117733 | 0 |
| 400 | LAKIE | IAQVV | 43.7215917 | 38.781776 | 48.6614072 | 0 |
| 400 | LAKIV | IAQVV | 1.8047387 | -3.135077 | 6.7445542 | 0.9363629 |
| 400 | LAKVV | IAQVV | -16.034684 | -20.9745 | -11.094869 | 0 |
| 400 | LAQVV | IAQVV | -4.832398 | -9.772213 | 0.1074176 | 0.0591016 |
| 400 | LVKIE | IAQVV | 52.0290936 | 47.089278 | 56.9689091 | 0 |
| 400 | Pikp-HMA | IAQVV | 50.6558776 | 45.716062 | 55.5956931 | 0 |
| 400 | LAKIV | LAKIE | -41.916853 | -46.856669 | -36.977038 | 0 |
| 400 | LAKVV | LAKIE | -59.756276 | -64.696092 | -54.81646 | 0 |
| 400 | LAQVV | LAKIE | -48.55399 | -53.493805 | -43.614174 | 0 |
| 400 | LVKIE | LAKIE | 8.3075019 | 3.367686 | 13.2473174 | 0.0000904 |
| 400 | Pikp-HMA | LAKIE | 6.9342859 | 1.99447 | 11.8741014 | 0.001416 |
| 400 | LAKVV | LAKIV | -17.839423 | -22.779238 | -12.899607 | 0 |
| 400 | LAQVV | LAKIV | -6.6371366 | -11.576952 | -1.6973211 | 0.0025111 |
| 400 | LVKIE | LAKIV | 50.2243549 | 45.284539 | 55.1641705 | 0 |
| 400 | Pikp-HMA | LAKIV | 48.8511389 | 43.911323 | 53.7909544 | 0 |
| 400 | LAQVV | LAKVV | 11.2022863 | 6.262471 | 16.1421019 | 0.0000002 |
| 400 | LVKIE | LAKVV | 68.0637779 | 63.123962 | 73.0035934 | 0 |
| 400 | Pikp-HMA | LAKVV | 66.6905618 | 61.750746 | 71.6303774 | 0 |
| 400 | LVKIE | LAQVV | 56.8614916 | 51.921676 | 61.8013071 | 0 |
| 400 | Pikp-HMA | LAQVV | 55.4882755 | 50.54846 | 60.4280911 | 0 |
| 400 | Pikp-HMA | LVKIE | -1.373216 | -6.313032 | 3.5665995 | 0.9854733 |

E230R: Pikp-HMA_E230R_, IAQVV: ancHMA_IAQVV_, LAQVV: ancHMA_LAQVV_, LAKVV: ancHMA_LAKVV_, LAKIV: ancHMA_LAKIV_, LAKIE: ancHMA_LAKIE_, LVKIE: ancHMA_LVKIE_

| Comparison | S_d_ | S_N_ | Total | S | N | p_S_ | p_N_ | *d*_S_ | *d*_N_ | *d*_S_/*d*_N_ |
| --- | --- | --- | --- | --- | --- | --- | --- | --- | --- | --- |
| Pikh vs. Pikp | 1 | 2 | 3 | 789.6667 | 2636.3333 | 0.0013 | 0.0008 | 0.0013 | 0.0008 | 1.6698 |
| Pikh vs. Pik* | 27.5 | 50.5 | 82 | 790.3333 | 2635.6667 | 0.0348 | 0.0192 | 0.0356 | 0.0194 | 1.8356 |
| Pikh vs. Pikm | 27.5 | 55.5 | 87 | 790.1667 | 2635.8333 | 0.0348 | 0.0211 | 0.0356 | 0.0214 | 1.6686 |
| Pikh vs. Piks | 27.5 | 54.5 | 86 | 790.1667 | 2635.8333 | 0.0348 | 0.0207 | 0.0356 | 0.021 | 1.6996 |
| Pikp vs. Pik* | 28.5 | 52.5 | 85 | 790.3333 | 2635.6667 | 0.0361 | 0.0199 | 0.037 | 0.0202 | 1.8306 |
| Pikp vs. Pikm | 28.5 | 57.5 | 90 | 790.1667 | 2635.8333 | 0.0361 | 0.0218 | 0.037 | 0.0221 | 1.6697 |
| Pikp vs. Piks | 28.5 | 56.5 | 89 | 790.1667 | 2635.8333 | 0.0361 | 0.0214 | 0.037 | 0.0217 | 1.6997 |
| Pik* vs. Pikm | 0 | 8 | 8 | 791.8333 | 2640.1667 | 0 | 0.003 | 0 | 0.003 | nan |
| Pik* vs. Piks | 0 | 8 | 8 | 791.8333 | 2640.1667 | 0 | 0.003 | 0 | 0.003 | nan |
| Pikm vs. Piks | 0 | 2 | 2 | 791.6667 | 2640.3333 | 0 | 0.0008 | 0 | 0.0008 | nan |

**Supplementary file 1J.** **Pairwise *d*_N_ and *d*_S_ values between Pik-1 alleles from rice calculated using the method of Nei and Gojobori (1986).**

S_d_: the number of observed synonymous substitutions

S_N_: the number of observed non-synonymous substitutions

S: the number of potential synonymous substitutions (the average for the two compared sequences)

N: the number of potential non-synonymous substitutions (the average for the two compared sequences)

P_S_: the proportion of observed synonymous substitutions: S_d_/S

p_N_: the proportion of observed non-synonymous substitutions: S_N_/N

*d*_S_: the Jukes-Cantor correction for multiple hits of p_S_

*d*_N_: the Jukes-Cantor correction for multiple hits of p_N_

*d*_S_/*d*_N_: The ratio of synonymous to non-synonymous substitutions

| Concentration  (nM) | Sample 1 | Sample 2 | Difference | Lower  confidence level | Upper  confidence level | p-value |
| --- | --- | --- | --- | --- | --- | --- |
| 400 | EMANK | E230R | 86.07761 | 62.02624 | 110.12898 | 0 |
| 400 | EMVKE | E230R | 91.209289 | 67.15792 | 115.26066 | 0 |
| 400 | MKANK | E230R | 83.519556 | 60.49213 | 106.54699 | 0 |
| 400 | Pikm | E230R | 77.298931 | 53.24757 | 101.3503 | 0 |
| 400 | EMVKE | EMANK | 5.131679 | -18.91969 | 29.18304 | 0.967442 |
| 400 | MKANK | EMANK | -2.558054 | -25.58548 | 20.46938 | 0.9971982 |
| 400 | Pikm | EMANK | -8.778679 | -32.83004 | 15.27269 | 0.8109631 |
| 400 | MKANK | EMVKE | -7.689733 | -30.71716 | 15.3377 | 0.8547095 |
| 400 | Pikm | EMVKE | -13.910358 | -37.96172 | 10.14101 | 0.4421083 |
| 400 | Pikm | MKANK | -6.220625 | -29.24805 | 16.8068 | 0.9262409 |
| 200 | EMANK | E230R | 65.82556 | 60.553591 | 71.09752 | 0 |
| 200 | EMVKE | E230R | 87.06222 | 81.790259 | 92.334189 | 0 |
| 200 | MKANK | E230R | 53.28956 | 48.017593 | 58.561522 | 0 |
| 200 | Pikm | E230R | 76.73675 | 71.464784 | 82.008713 | 0 |
| 200 | EMVKE | EMANK | 21.23667 | 15.964704 | 26.508633 | 0 |
| 200 | MKANK | EMANK | -12.536 | -17.807963 | -7.264034 | 0.0000209 |
| 200 | Pikm | EMANK | 10.91119 | 5.639228 | 16.183157 | 0.0001023 |
| 200 | MKANK | EMVKE | -33.77267 | -39.044631 | -28.500702 | 0 |
| 200 | Pikm | EMVKE | -10.32548 | -15.59744 | -5.053511 | 0.0001865 |
| 200 | Pikm | MKANK | 23.44719 | 18.175226 | 28.719156 | 0 |
| 50 | EMANK | E230R | 38.83454 | 21.734495 | 55.934593 | 0.0000044 |
| 50 | EMVKE | E230R | 76.07891 | 58.978857 | 93.178955 | 0 |
| 50 | MKANK | E230R | 27.8971 | 10.797048 | 44.997145 | 0.0005356 |
| 50 | Pikm | E230R | 63.94274 | 46.842695 | 81.042793 | 0 |
| 50 | EMVKE | EMANK | 37.24436 | 19.498803 | 54.989921 | 0.0000157 |
| 50 | MKANK | EMANK | -10.93745 | -28.683007 | 6.808111 | 0.3922477 |
| 50 | Pikm | EMANK | 25.1082 | 7.362641 | 42.853759 | 0.0027261 |
| 50 | MKANK | EMVKE | -48.18181 | -65.927369 | -30.436251 | 0.0000002 |
| 50 | Pikm | EMVKE | -12.13616 | -29.881721 | 5.609397 | 0.2926768 |
| 50 | Pikm | MKANK | 36.04565 | 18.300089 | 53.791207 | 0.000026 |

**Supplementary file 1K. Table of p-values for all pairwise comparisons of SPR binding to AVR-PikD between the HMA mutants.**

E230R: Pikp-HMA_E230R_, EMVKE: ancHMA_EMVKE_, EMANK: ancHMA_EMANK_, MKANK: ancHMA_;_ Pikm: Pikm-HMA

**Supplementary file 1L. Data collection and refinement statistics for the ancHMA_LVKIE_–AVR-PikD co-crystal structure.**

|  | | Value |
| --- | --- | --- |
| Data collection statistics | |  |
|  | Beamline | I03 Diamond |
|  | Detector | Pilatus3 6M |
|  | Wavelength (Å) | 0.9700 |
|  | Space group | *P* 4_1_2_1_2 |
|  | Cell dimensions (Å) | *a* = *b* = 119.5, c = 36.0 |
|  | Resolution (Å)* | 59.81–1.32 (1.34–1.32) |
|  | *R*_merge_^#^ | 0.068 (2.201) |
|  | *R*_meas_^#^ | 0.070 (2.285) |
|  | Mean *I*/σ(*I)*# | 16.7 (1.3) |
|  | *CC_(1/2)_^#^* | 0.999 (0.730) |
|  | Completeness (%)^#^ | 96.3 (94.1) |
|  | Unique reflections^#^ | 59,464 (2,862) |
|  | Multiplicity^#^ | 14.6 (13.9) |
|  | Wilson *B* value (Å^2^)^#^ | 16.0 |
| Refinement and model statistics | |  |
|  | Resolution (Å) | 59.81-1.32 (1.35-1.32) |
|  | *R*work^†^ | 0.145 (0.271) |
|  | *R*free^†^ | 0.184 (0.295) |
|  | Mean *B* factors: protein/waters/overall (Å^2^) | 22/35/24 |
|  | R.m.s. bond deviations (Å)^†^ | 0.011 |
|  | R.m.s. angle deviations (°)^†^ | 1.59 |
|  | Ramachandran plot: favoured/allowed/outliers(%)** | 98.2/1.8/0.0 |
|  | MolProbity Score | 1.09 |
| PDB Accession code | | 7BNT |

*the highest resolution shell is shown in parentheses

**as calculated by MolProbity, ^#^as calculated by Aimless

^†^ as calculated by REFMAC5

| Description on the tree | Species | Accession number | Pik-integrated | used for ASR | | |
| --- | --- | --- | --- | --- | --- | --- |
|  |  |  |  | I | II | III |
| *O.barthii*_W0042 | *O. barthii* | MW553215 | y | n | n | y |
| *O.barthii*_W1643 | *O. barthii* | MW553205 | y | n | n | y |
| *O.punctata*_W1408 | *O. punctata* | MW553208 | y | n | n | y |
| *O.barthii*_W0698 | *O. barthii* | MW553211 | y | n | n | y |
| *O.granulata*_W0067B | *O. granulata* | MW553214 | y | n | n | y |
| *O.longistaminata*_W0643 | *O. longistaminata* | MW553212 | y | n | n | y |
| *O.officinalis*_W0614 | *O. officinalis* | MW553213 | y | n | n | y |
| *O.punctata*_W1514 | *O. punctata* | MW553207 | y | n | n | y |
| *O.rufipogon*_W2003 | *O. rufipogon* | MW553204 | y | n | n | y |
| *O.minuta*_W1328 | *O. minuta* | MW553209 | y | n | n | y |
| LOC102699268 | *O. brachyantha* | LOC102699268 | y | y | y | y |
| OBART11G23150 | *O. barthii* | OBART11G23150 | y | n | n | y |
| *Olongi*_KN541092.1 | *O. longistaminata* | KN541092.1 | y | n | n | y |
| OPUNC11G19550 | *O. punctata* | OPUNC11G19550 | y | n | n | y |
| *Os*Pikp-1 | *O. sativa* | HM035360.1 | y | y | y | y |
| *Os*Pik*-1 | *O. sativa* | HM048900_1 | y | y | y | Y |
| *Os*Pikh-1 | *O. sativa* | HQ662330_1 | y | y | y | Y |
| *Os*Piks-1 | *O. sativa* | HQ662329_1 | y | y | y | y |
| OsPikm-1 | *O. sativa* | AB462324.1 | y | y | y | y |
| Ob_LOC102708959 | *O. brachyantha* | LOC102708959 | n | n | y | y |
| Ob_LOC102709146 | *O. brachyantha* | LOC102709146 | n | n | y | y |
| Ob_LOC102714171 | *O. brachyantha* | LOC102714171 | n | n | y | y |
| Ob_LOC102716957 | *O. brachyantha* | LOC102716957 | n | n | y | y |
| Ob_LOC102717220 | *O. brachyantha* | LOC102717220 | n | n | y | y |
| Os_LOC_Os04g39360 | *O. sativa* | LOC_Os04g39360 | n | y | y | y |
| Os_LOC_Os04g39370 | *O. sativa* | LOC_Os04g39370 | n | y | y | y |
| Os04g0469000_01 | *O. sativa* | Os04g0469000_01 | n | y | y | y |
| Os02g0585200 | *O. sativa* | Os02g0585200 | n | y | y | y |
| Os02g0584800_01 | *O. sativa* | Os02g0584800_01 | n | y | y | y |
| Os02g0584700_01 | *O. sativa* | Os02g0584700_01 | n | y | y | y |
| Os04g0469300_01 | *O. sativa* | Os04g0469300_01 | n | y | y | y |
| Os02g0585100 | *O. sativa* | Os02g0585100 | n | y | y | y |
| Os02g0584600 | *O. sativa* | Os02g0584600 | n | y | y | y |
| OSJNBa0060P14.7_01 | *O. sativa* | OSJNBa0060P14.7_01 | n | y | y | y |
| Os04g0464100_01 | *O. sativa* | Os04g0464100_01 | n | y | y | y |
| Os02g0582600 | *O. sativa* | Os02g0582600 | n | y | y | y |

**Supplementary file 1M. HMA sequences used for building phylogenetic trees and ancestral sequence reconstruction (ASR).**

| Protein name | Tag | Vector backbone | Concentration (OD600) | Reference |
| --- | --- | --- | --- | --- |
| Pikp-1 | 6×HA (N-term) | pICH77732 | 0.15 | This study |
| Pikp-1^+^ | 6×HA (N-term) | pICH77732 | 0.15 | This study |
| Pikp-2 | 3×Myc (C-term) | pCambia | 0.15 | Maqbool et al., 2015 |
| AVR-PikD | 3×FLAG (C-term) | pTRBO | 0.15 | This study |
| AVRblb2 | 3×FLAG (C-term) | pTRBO | 0.15 | Bozkurt et al., 2011 |
| Pikp-1 | 6×His/ 3×FLAG (HF) (C-term) | pICH47742 | 0.4 | De la Concepcion et al., 2018 |
| Pikp-1 | 6×His/ 3×FLAG (HF) (C-term) | pICH47732 | 0.4 | This study |
| Pikp-2 | 6×HA (C-term) | pICH47751 | 0.4 | De la Concepcion et al., 2018 |
| AVR-PikD | 4×Myc (N-term) | pICH47732 | 0.6 | De la Concepcion et al., 2018 |
| P19 | NA | pCB301 | 0.1 | Win and Kamoun, 2003 |
| Pikm-1 | 6×His/ 3×FLAG (C-term) | pICH47742 | 0.4 | De la Concepcion et al., 2018 |
| Pikm-1^+^ | 6×His/ 3×FLAG (C-term) | pICH47732 | 0.4 | This study |
| Pikm-2 | 6×HA (C-term) | pICH47751 | 0.4 | De la Concepcion et al., 2018 |

**Supplementary file 1N. List of constructs used in cell death assays.**

^+^including mutants and fusions

NA: not applicable

**References**

Bennetzen JL, Schmutz J, Wang H, Percifield R, Hawkins J, Pontaroli AC, Estep M, Feng L, Vaughn JN, Grimwood J, Jenkins J, Barry K, Lindquist E, Hellsten U, Deshpande S, Wang X, Wu X, Mitros T, Triplett J, Yang X, Ye CY, Mauro-Herrera M, Wang L, Li P, Sharma M, Sharma R, Ronald PC, Panaud O, Kellogg EA, Brutnell TP, Doust AN, Tuskan GA, Rokhsar D, Devos KM. 2012. Reference genome sequence of the model plant setaria. Nature Biotechnology **30**:555–561.

Bhullar NK, Zhang Z, Wicker T, Keller B. 2010. Wheat gene bank accessions as a source of new alleles of the powdery mildew resistance gene Pm3: a large scale allele mining project. BMC Plant Biology **10**:88.

Bozkurt TO, Schornack S, Win J, Shindo T, Ilyas M, Oliva R, Cano LM, Jones AM, Huitema E, van der Hoorn RA, Kamoun S. 2011. Phytophthora infestans effector AVRblb2 prevents secretion of a plant immune protease at the haustorial interface. PNAS **108**:20832–20837.

Brueggeman R, Druka A, Nirmala J, Cavileer T, Drader T, Rostoks N, Mirlohi A, Bennypaul H, Gill U, Kudrna D, Whitelaw C, Kilian A, Han F, Sun Y, Gill K, Steffenson B, Kleinhofs A. 2008. The stem rust resistance gene Rpg5 encodes a protein with nucleotide-binding-site, leucine-rich, and protein kinase domains. PNAS **105**:14970–14975.

Bryan GT, Wu KS, Farrall L, Jia Y, Hershey HP, McAdams SA, Faulk KN, Donaldson GK, Tarchini R, Valent B. 2000. tA single amino acid difference distinguishes resistant and susceptible alleles of the rice blast resistance gene Pi-ta. The Plant Cell **12**:2033–2045.

Bulgarelli D, Biselli C, Collins NC, Consonni G, Stanca AM, Schulze-Lefert P, Valè G. 2010. The CC-NB-LRR-type Rdg2a resistance gene confers immunity to the seed-borne barley leaf stripe pathogen in the absence of hypersensitive cell death. PLOS ONE **5**:e12599.

Collins N, Drake J, Ayliffe M, Sun Q, Ellis J, Hulbert S, Pryor T. 1999. Molecular characterization of the maize Rp1-D rust resistance haplotype and its mutants. The Plant Cell **11**:1365–1376.

Feuillet C, Travella S, Stein N, Albar L, Nublat A, Keller B. 2003. Map-based isolation of the leaf rust disease resistance gene Lr10 from the hexaploid wheat (Triticum aestivum L.) genome. PNAS **100**:15253–15258.

Halterman DA, Wise RP. 2004. A single-amino acid substitution in the sixth leucine-rich repeat of barley MLA6 and MLA13 alleviates dependence on RAR1 for disease resistance signaling. The Plant Journal **38**:215–226.

Hayashi K, Yoshida H. 2009. Refunctionalization of the ancient rice blast disease resistance gene pit by the recruitment of a retrotransposon as a promoter. The Plant Journal **57**:413–425.

Huang L, Brooks S, Li W, Fellers J, Nelson JC, Gill B. 2009. Evolution of new disease specificity at a simple resistance locus in a crop-weed complex: reconstitution of the Lr21 gene in wheat. Genetics **182**:595–602.

Huang L, Feng G, Yan H, Zhang Z, Bushman BS, Wang J, Bombarely A, Li M, Yang Z, Nie G, Xie W, Xu L, Chen P, Zhao X, Jiang W, Zhang X. 2020. Genome assembly provides insights into the genome evolution and flowering regulation of orchardgrass. Plant Biotechnology Journal **18**:373–388.

Hurni S, Brunner S, Buchmann G, Herren G, Jordan T, Krukowski P, Wicker T, Yahiaoui N, Mago R, Keller B. 2013. Rye Pm8 and wheat Pm3 are orthologous genes and show evolutionary conservation of resistance function against powdery mildew. The Plant Journal : For Cell and Molecular Biology **76**:957–969.

International Brachypodium Initiative. 2010. Genome sequencing and analysis of the model grass Brachypodium distachyon. Nature **463**:763–768.

International Wheat Genome Sequencing Consortium (IWGSC), IWGSC RefSeq principal investigators:, IWGSC whole-genome assembly principal investigators:, Whole-genome sequencing and assembly:, Hi-C data-based scaffolding:, Whole-genome assembly quality control and analyses:, Pseudomolecule assembly:, RefSeq genome structure and gene analyses:, Automated annotation:, Manual gene curation:, Subgenome comparative analyses:, Transposable elements:, Phylogenomic analyses:, Transcriptome analyses and RNA-seq data:, Whole-genome methylome:, Histone mark analyses:, BAC chromosome MTP IWGSC–Bayer Whole-Genome Profiling (WGP) tags:, Chromosome LTC mapping and physical mapping quality control:, RH mapping:, Optical mapping:, Recombination analyses:, Gene family analyses:, CBF gene family:, Dehydrin gene family:, NLR gene family:, PPR gene family:, Prolamin gene family:, WAK gene family:, Stem solidness (SSt1) QTL team:, Flowering locus C (FLC) gene team:, Genome size analysis:, MicroRNA and tRNA annotation:, Genetic maps and mapping:, BAC libraries and chromosome sorting:, BAC pooling, BAC library repository, and access:, IWGSC sequence and data repository and access:, Physical maps and BAC-based sequences:, 1A BAC sequencing and assembly:, 1B BAC sequencing and assembly:, 1D, 4D, and 6D physical mapping:, 2AL physical mapping:, 2AS physical mapping:, 2B, 2D, 4B, 5BL, and 5DL IWGSC–Bayer Whole-Genome Profiling (WGP) physical maps:, 3AL physical mapping:, 3DS physical mapping and BAC sequencing and assembly:, 3DL BAC sequencing and assembly:, 4A physical mapping, BAC sequencing, assembly, and annotation:, 5BS BAC sequencing and assembly:, 6B BAC sequencing and assembly:, 7A physical mapping and BAC sequencing:, 7B physical mapping, BAC sequencing, and assembly:, 7DS BAC sequencing and assembly:, 7DL physical mapping and BAC sequencing:, Figures:, Manuscript writing team:, Appels R, Eversole K, Feuillet C, Keller B, Rogers J, Stein N, Pozniak CJ, Stein N, Choulet F, Distelfeld A, Eversole K, Poland J, Rogers J, Ronen G, Sharpe AG, Pozniak C, Ronen G, Stein N, Barad O, Baruch K, Choulet F, Keeble-Gagnère G, Mascher M, Sharpe AG, Ben-Zvi G, Josselin AA, Stein N, Mascher M, Himmelbach A, Choulet F, Keeble-Gagnère G, Mascher M, Rogers J, Balfourier F, Gutierrez-Gonzalez J, Hayden M, Josselin AA, Koh C, Muehlbauer G, Pasam RK, Paux E, Pozniak CJ, Rigault P, Sharpe AG, Tibbits J, Tiwari V, Choulet F, Keeble-Gagnère G, Mascher M, Josselin AA, Rogers J, Spannagl M, Choulet F, Lang D, Gundlach H, Haberer G, Keeble-Gagnère G, Mayer KFX, Ormanbekova D, Paux E, Prade V, Šimková H, Wicker T, Choulet F, Spannagl M, Swarbreck D, Rimbert H, Felder M, Guilhot N, Gundlach H, Haberer G, Kaithakottil G, Keilwagen J, Lang D, Leroy P, Lux T, Mayer KFX, Twardziok S, Venturini L, Appels R, Rimbert H, Choulet F, Juhász A, Keeble-Gagnère G, Choulet F, Spannagl M, Lang D, Abrouk M, Haberer G, Keeble-Gagnère G, Mayer KFX, Wicker T, Choulet F, Wicker T, Gundlach H, Lang D, Spannagl M, Lang D, Spannagl M, Appels R, Fischer I, Uauy C, Borrill P, Ramirez-Gonzalez RH, Appels R, Arnaud D, Chalabi S, Chalhoub B, Choulet F, Cory A, Datla R, Davey MW, Hayden M, Jacobs J, Lang D, Robinson SJ, Spannagl M, Steuernagel B, Tibbits J, Tiwari V, van Ex F, Wulff BBH, Pozniak CJ, Robinson SJ, Sharpe AG, Cory A, Benhamed M, Paux E, Bendahmane A, Concia L, Latrasse D, Rogers J, Jacobs J, Alaux M, Appels R, Bartoš J, Bellec A, Berges H, Doležel J, Feuillet C, Frenkel Z, Gill B, Korol A, Letellier T, Olsen OA, Šimková H, Singh K, Valárik M, van der Vossen E, Vautrin S, Weining S, Korol A, Frenkel Z, Fahima T, Glikson V, Raats D, Rogers J, Tiwari V, Gill B, Paux E, Poland J, Doležel J, Číhalíková J, Šimková H, Toegelová H, Vrána J, Sourdille P, Darrier B, Appels R, Spannagl M, Lang D, Fischer I, Ormanbekova D, Prade V, Barabaschi D, Cattivelli L, Hernandez P, Galvez S, Budak H, Steuernagel B, Jones JDG, Witek K, Wulff BBH, Yu G, Small I, Melonek J, Zhou R, Juhász A, Belova T, Appels R, Olsen OA, Kanyuka K, King R, Nilsen K, Walkowiak S, Pozniak CJ, Cuthbert R, Datla R, Knox R, Wiebe K, Xiang D, Rohde A, Golds T, Doležel J, Čížková J, Tibbits J, Budak H, Akpinar BA, Biyiklioglu S, Muehlbauer G, Poland J, Gao L, Gutierrez-Gonzalez J, N'Daiye A, Doležel J, Šimková H, Číhalíková J, Kubaláková M, Šafář J, Vrána J, Berges H, Bellec A, Vautrin S, Alaux M, Alfama F, Adam-Blondon AF, Flores R, Guerche C, Letellier T, Loaec M, Quesneville H, Pozniak CJ, Sharpe AG, Walkowiak S, Budak H, Condie J, Ens J, Koh C, Maclachlan R, Tan Y, Wicker T, Choulet F, Paux E, Alberti A, Aury JM, Balfourier F, Barbe V, Couloux A, Cruaud C, Labadie K, Mangenot S, Wincker P, Gill B, Kaur G, Luo M, Sehgal S, Singh K, Chhuneja P, Gupta OP, Jindal S, Kaur P, Malik P, Sharma P, Yadav B, Singh NK, Khurana J, Chaudhary C, Khurana P, Kumar V, Mahato A, Mathur S, Sevanthi A, Sharma N, Tomar RS, Rogers J, Jacobs J, Alaux M, Bellec A, Berges H, Doležel J, Feuillet C, Frenkel Z, Gill B, Korol A, van der Vossen E, Vautrin S, Gill B, Kaur G, Luo M, Sehgal S, Bartoš J, Holušová K, Plíhal O, Clark MD, Heavens D, Kettleborough G, Wright J, Valárik M, Abrouk M, Balcárková B, Holušová K, Hu Y, Luo M, Salina E, Ravin N, Skryabin K, Beletsky A, Kadnikov V, Mardanov A, Nesterov M, Rakitin A, Sergeeva E, Handa H, Kanamori H, Katagiri S, Kobayashi F, Nasuda S, Tanaka T, Wu J, Appels R, Hayden M, Keeble-Gagnère G, Rigault P, Tibbits J, Olsen OA, Belova T, Cattonaro F, Jiumeng M, Kugler K, Mayer KFX, Pfeifer M, Sandve S, Xun X, Zhan B, Šimková H, Abrouk M, Batley J, Bayer PE, Edwards D, Hayashi S, Toegelová H, Tulpová Z, Visendi P, Weining S, Cui L, Du X, Feng K, Nie X, Tong W, Wang L, Borrill P, Gundlach H, Galvez S, Kaithakottil G, Lang D, Lux T, Mascher M, Ormanbekova D, Prade V, Ramirez-Gonzalez RH, Spannagl M, Stein N, Uauy C, Venturini L, Stein N, Appels R, Eversole K, Rogers J, Borrill P, Cattivelli L, Choulet F, Hernandez P, Kanyuka K, Lang D, Mascher M, Nilsen K, Paux E, Pozniak CJ, Ramirez-Gonzalez RH, Šimková H, Small I, Spannagl M, Swarbreck D, Uauy C. 2018. Shifting the limits in wheat research and breeding using a fully annotated reference genome. Science **361**:eaar7191.

Kawahara Y, Bastide M, Hamilton JP, Kanamori H, McCombie WR, Ouyang S, Schwartz DC, Tanaka T, Wu J, Zhou S, Childs KL, Davidson RM, Lin H, Quesada-Ocampo L, Vaillancourt B, Sakai H, Lee SS, Kim J, Numa H, Itoh T, Buell CR, Matsumoto T. 2013. Improvement of the Oryza sativa nipponbare reference genome using next generation sequence and optical map data. Rice **6**:4.

Lee SK, Song MY, Seo YS, Kim HK, Ko S, Cao PJ, Suh JP, Yi G, Roh JH, Lee S, An G, Hahn TR, Wang GL, Ronald P, Jeon JS. 2009. Rice Pi5-mediated resistance to Magnaporthe oryzae requires the presence of two coiled-coil-nucleotide-binding-leucine-rich repeat genes. Genetics **181**:1627–1638.

Liu W, Frick M, Huel R, Nykiforuk CL, Wang X, Gaudet DA, Eudes F, Conner RL, Kuzyk A, Chen Q, Kang Z, Laroche A. 2014. The stripe rust resistance gene Yr10 encodes an evolutionary-conserved and unique CC-NBS-LRR sequence in wheat. Molecular Plant **7**:1740–1755.

Mago R, Zhang P, Vautrin S, Šimková H, Bansal U, Luo MC, Rouse M, Karaoglu H, Periyannan S, Kolmer J, Jin Y, Ayliffe MA, Bariana H, Park RF, McIntosh R, Doležel J, Bergès H, Spielmeyer W, Lagudah ES, Ellis JG, Dodds PN. 2015. The wheat Sr50 gene reveals rich diversity at a cereal disease resistance locus. Nature Plants **1**:15186.

Mascher M, Gundlach H, Himmelbach A, Beier S, Twardziok SO, Wicker T, Radchuk V, Dockter C, Hedley PE, Russell J, Bayer M, Ramsay L, Liu H, Haberer G, Zhang XQ, Zhang Q, Barrero RA, Li L, Taudien S, Groth M, Felder M, Hastie A, Šimková H, Staňková H, Vrána J, Chan S, Muñoz-Amatriaín M, Ounit R, Wanamaker S, Bolser D, Colmsee C, Schmutzer T, Aliyeva-Schnorr L, Grasso S, Tanskanen J, Chailyan A, Sampath D, Heavens D, Clissold L, Cao S, Chapman B, Dai F, Han Y, Li H, Li X, Lin C, McCooke JK, Tan C, Wang P, Wang S, Yin S, Zhou G, Poland JA, Bellgard MI, Borisjuk L, Houben A, Doležel J, Ayling S, Lonardi S, Kersey P, Langridge P, Muehlbauer GJ, Clark MD, Caccamo M, Schulman AH, Mayer KFX, Platzer M, Close TJ, Scholz U, Hansson M, Zhang G, Braumann I, Spannagl M, Li C, Waugh R, Stein N. 2017. A chromosome conformation capture ordered sequence of the barley genome. Nature **544**:427–433.

McCoy AJ, Grosse-Kunstleve RW, Adams PD, Winn MD, Storoni LC, Read RJ. 2007. Phaser crystallographic software. Journal of Applied Crystallography **40**:658–674.

Paterson AH, Bowers JE, Bruggmann R, Dubchak I, Grimwood J, Gundlach H, Haberer G, Hellsten U, Mitros T, Poliakov A, Schmutz J, Spannagl M, Tang H, Wang X, Wicker T, Bharti AK, Chapman J, Feltus FA, Gowik U, Grigoriev IV, Lyons E, Maher CA, Martis M, Narechania A, Otillar RP, Penning BW, Salamov AA, Wang Y, Zhang L, Carpita NC, Freeling M, Gingle AR, Hash CT, Keller B, Klein P, Kresovich S, McCann MC, Ming R, Peterson DG, Rahman M, Ware D, Westhoff P, Mayer KF, Messing J, Rokhsar DS. 2009. The Sorghum bicolor genome and the diversification of grasses. Nature **457**:551–556.

Qu S, Liu G, Zhou B, Bellizzi M, Zeng L, Dai L, Han B, Wang GL. 2006. The broad-spectrum blast resistance gene Pi9 encodes a nucleotide-binding site-leucine-rich repeat protein and is a member of a multigene family in rice. Genetics **172**:1901–1914.

Shang J, Tao Y, Chen X, Zou Y, Lei C, Wang J, Li X, Zhao X, Zhang M, Lu Z, Xu J, Cheng Z, Wan J, Zhu L. 2009. Identification of a new rice blast resistance gene, Pid3, by genomewide comparison of paired nucleotide-binding site--leucine-rich repeat genes and their pseudogene alleles between the two sequenced rice genomes. Genetics **182**:1303–1311.

Steuernagel B, Periyannan SK, Hernández-Pinzón I, Witek K, Rouse MN, Yu G, Hatta A, Ayliffe M, Bariana H, Jones JD, Lagudah ES, Wulff BB. 2016. Rapid cloning of disease-resistance genes in plants using mutagenesis and sequence capture. Nature Biotechnology **34**:652–655.

Thind AK, Wicker T, Šimková H, Fossati D, Moullet O, Brabant C, Vrána J, Doležel J, Krattinger SG. 2017. Rapid cloning of genes in hexaploid wheat using cultivar-specific long-range chromosome assembly. Nature Biotechnology **35**:793–796.

Wang ZX, Yano M, Yamanouchi U, Iwamoto M, Monna L, Hayasaka H, Katayose Y, Sasaki T. 1999. The pib gene for rice blast resistance belongs to the nucleotide binding and leucine-rich repeat class of plant disease resistance genes. The Plant Journal **19**:55–64.

Yoshimura S, Yamanouchi U, Katayose Y, Toki S, Wang ZX, Kono I, Kurata N, Yano M, Iwata N, Sasaki T. 1998. Expression of Xa1, a bacterial blight-resistance gene in rice, is induced by bacterial inoculation. PNAS **95**:1663–1668.

Yuan B, Zhai C, Wang W, Zeng X, Xu X, Hu H, Lin F, Wang L, Pan Q. 2011. The Pik-p resistance to Magnaporthe oryzae in rice is mediated by a pair of closely linked CC-NBS-LRR genes. Theoretical and Applied Genetics **122**:1017–1028.

Zhang L, Chia JM, Kumari S, Stein JC, Liu Z, Narechania A, Maher CA, Guill K, McMullen MD, Ware D. 2009. A genome-wide characterization of microRNA genes in maize. PLOS Genetics **5**:e1000716.

Zhou B, Qu S, Liu G, Dolan M, Sakai H, Lu G, Bellizzi M, Wang GL. 2006. The eight amino-acid differences within three leucine-rich repeats between Pi2 and Piz-t resistance proteins determine the resistance specificity to Magnaporthe grisea. Molecular Plant-Microbe Interactions **19**:1216–1228.
